# Chemical Composition of Herbal Macerates and Corresponding Commercial Essential Oils and Their Effect on Bacteria *Escherichia coli*

**DOI:** 10.3390/molecules22111887

**Published:** 2017-11-10

**Authors:** Marietta Białoń, Teresa Krzyśko-Łupicka, Agnieszka Pik, Piotr P. Wieczorek

**Affiliations:** 1 Faculty of Chemistry, University of Opole, Oleska 48, 45-052 Opole, Poland; agnieszka.pik@op.pl (A.P.); pwiecz@uni.opole.pl (P.P.W.); 2 Independent Department of Biotechnology and Molecular Biology, Faculty of Natural and Technical Science, University of Opole, Kominka 6A, 45-035 Opole, Poland; teresak@uni.opole.pl

**Keywords:** essential oils, gas chromatography-mass spectrometry, *Escherichia coli*

## Abstract

This study addresses the chemical composition of some commercial essential oils (clove, juniper, oregano, and marjoram oils), as well as appropriate herbal extracts obtained in the process of cold maceration and their biological activity against selected *Escherichia coli* strains: *E. coli* ATTC 25922, *E. coli* ATTC 10536, and *E. coli* 127 isolated from poultry waste. On the basis of the gas chromatography-mass spectrometry (GCMS) analysis, it was found that the commercial essential oils revealed considerable differences in terms of the composition and diversity of terpenes, terpenoids and sesquiterpenes as compared with the extracts obtained from plant material. The commercial clove, oregano, and marjoram oils showed antibacterial properties against all the tested strains of *E. coli*. However, these strains were not sensitive to essential oils obtained from the plant material in the process of maceration. The tested strains of *E. coli* show a high sensitivity, mainly against monoterpenes (*α*-pinene, *β*-pinene, *α*,*β*,*γ*-terpinene, limonene) and some terpenoids (thymol, carvacrol). The commercial juniper oil contained mainly monoterpenes and monoterpenoids, while the extracts contained lower amounts of monoterpenes and high amounts of sesquiterpenes—the anti-microbiotic properties of the juniper herbal extract seem to be caused by the synergistic activity of mono- and sesquiterpenes.

## 1. Introduction

Oils or oily plants are used as spices, therapeutic agents, in herbal medicine and aromatherapy as well as flavouring components of perfume or toilet water in the cosmetics industry. Due to their disinfectant properties, some oils are used in the food industry and restaurant to disinfect potable water and preserve food, as well as in the cultivation of plants and tending pets [1].

In recent years, due to the high survivability of microorganisms in the environment caused by resistance to antibiotics and preservative agents, the natural anti-microbiological preparations, such as extracts and essential oils, have become the centre of attention [2].

Depending on the chemical composition, essential oils and extracts can be divided into terpene and non-terpene oils. Terpene oils contain mainly terpenes, most frequently mono-, sesqui-, and less frequently, di-terpenes, and the non-terpene oils contain phenylpropane derivatives. Compounds found in both of these oil groups are present, for example, in the form of hydrocarbons, alcohols, ketones, aldehydes, phenols, esters, and acids. Some oils may also contain compounds containing sulphur, nitrogen, and coumarins [3].

The efficacy of antimicrobial action of oils and extracts depends to a great degree on the chemical composition, which is connected with the type of oily plants, the growth conditions, harvesting, and processing, as well as the manner of obtaining the extracts. Differences in the chemical composition and biological activity were observed in garlic oils containing sulphur compounds [4,5,6,7], nettle [8,9,10], and chamomile [11], which contain biogenic amines and angelica-containing coumarins [12,13,14]. Furthermore, what is also significant is the qualitative-quantitative composition of various chemical compounds found in essential oils, since it is responsible for their antimicrobial efficacy.

Clove oil is an extremely important substance which inhibits the growth of the bacteria (e.g., *Escherichia coli*, *Bacillus subtilis*), probably thanks to the presence of eugenol, eugenol acetate, and caryophyllene [15,16], and so is oregano oil, perhaps due to the presence of carvacrol [17,18]. On the other hand, *α*- and *β*-pinene present in juniper and marjoram oil and, additionally, limonene in marjoram oil may be responsible for the anti-viral, anti-fungal, and antibacterial properties [19,20,21,22].

The aim of the paper is to compare the chemical composition of chosen commercial oils with extracts obtained from plant raw material (spices) in the process of cold maceration and hot extraction in the Soxhlet apparatus, as well as to determine their biological activity against the *E. coli* strains.

## 2. Results

Regardless of the applied procedure, the extraction efficiency of the tested plant raw material was similar (Table 1).

**Figure 1:**
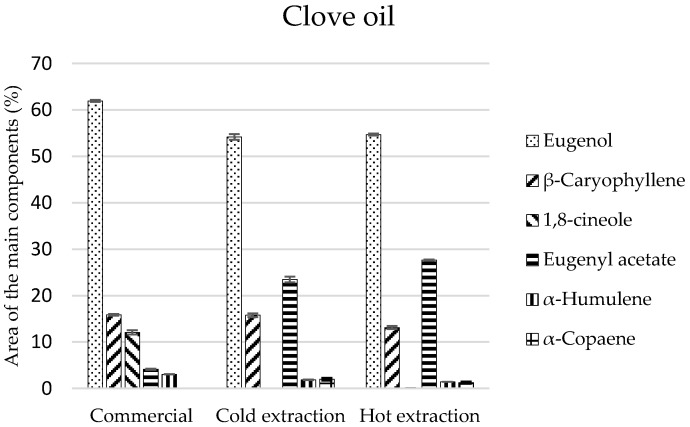
The composition of the main components in the clove commercial oil and extracts obtained in the process of cold and hot extraction.

The tested oils revealed a diversified qualitative-quantitative composition. In clove oil, a different number of chemical compounds was identified: the most in the commercial oil (20 components), and the least in cold and hot extracts, 14 and 13 components, respectively (Table 2). Monoterpenes constituted the main components of these oils and in the commercial oil and after maceration they amounted to 78% and, after hot extraction, 82% (Table 2). Eugenol was the most important part of this group of compounds, which made up 62% of the whole composition while, in the remaining extracts, it comprised approximately 55%. However, eugenol acetate was present in considerable amounts (23.5% and 27.5%) only in extracts (Figure 1). The sesquiterpene-type compounds constituted around 20% of the commercial oil and maceration-obtained compositions, while 16% of the composition of the extract obtained as the result of extraction in the Soxhlet apparatus. In this group of compounds, *β*-caryophyllene was observed in the highest amounts (16% and 13%). On the other hand, monoterpenes, which were mostly represented by eucalyptol, were present only in the commercial oil (Table 2).

**Figure 2:**
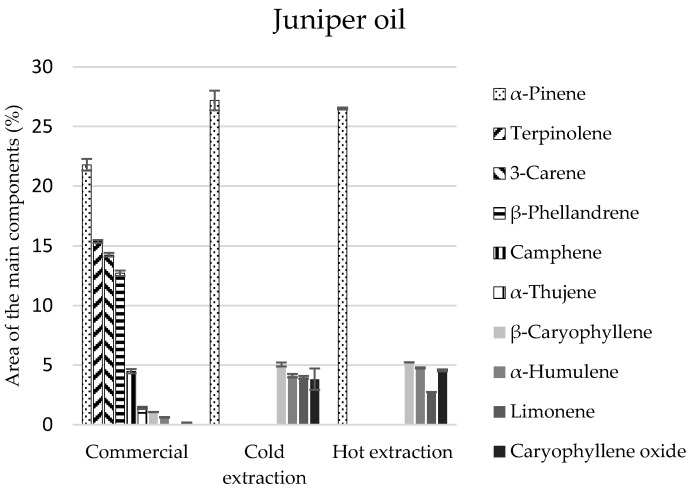
The composition of the main components in the juniper commercial oil and extracts obtained in the process of cold and hot extraction.

In the commercial juniper oil, there were 38 identified components, and in the extracted oils, 23 in each of the extracts (Table 3). Monoterpenes constituted 88% of the commercial juniper oil composition and in the extracts their amount was two times lower and equalled 45%, on average. The second group of compounds in extracts, regardless of the manner of obtaining them, was sesquiterpenes in the amount of 21%, on average (Table 3). *α*-Pinene was the main component to be found in the greatest amounts in all the tested juniper oils (approximately 22%) (Figure 2). However, these oils differed in terms of the quantitative-qualitative composition of the remaining components. Terpinolene, 3-carene, *β*-phellandrene of the monoterpene group were identified only in the commercial oil, and the extracts contained *β*-thujene and sesquiterpenes in considerable amounts (*β*-caryophyllene, and *α*-humulene).

**Figure 3:**
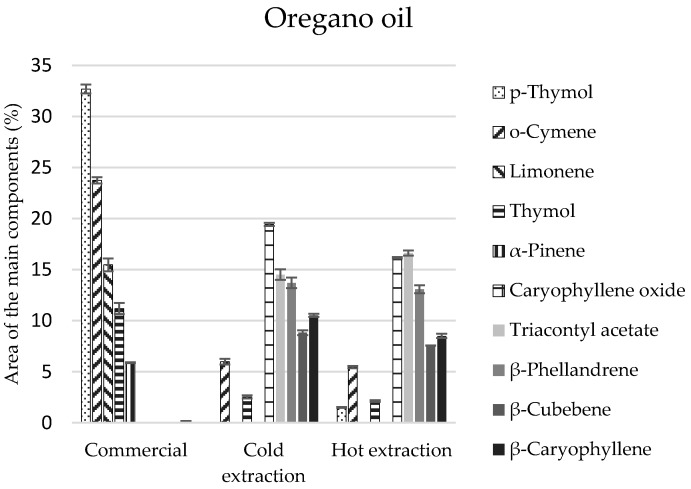
The composition of the main components in the oregano commercial oil and extracts obtained in the process of cold and hot extraction.

Eighteen compounds were identified in the commercial oregano oil, and more in extracts (21 and 23 components) (Table 4). The commercial oregano oil contained monoterpenes in comparable amounts (51%) and their oxygen derivatives (49%) (Table 4). Compositions of oils obtained by the extraction methods were differentiated and different from the composition of the commercial oregano oil—monoterpenes and their derivatives constituted 36% each, and sesquiterpenes and sesquiterpenoids constituted 40–50% (Table 4). *p*-Thymol and thymol of the monoterpenoid group constituted the main components of the commercial oil (total of 43.9%) (Figure 3). Their presence in extracts obtained in the cold and hot manner was significantly lower and equalled 2.5% and 3.6%, respectively (Table 4). However, the main elements of extracts included the following: caryophyllene, triacontyl acetate, and *β*-phellandrene (Figure 3), which were not identified in the commercial oil. On the other hand, carvacrol, a compound revealing biocidal properties, was identified only in the cold extract, in the amount of 1.8% (Table 4), although it is enumerated as one of the main components of commercial oils. The lack of this compound can be caused by genetic changes in the plants, as well as the environmental conditions in oregano plantations.

**Figure 4:**
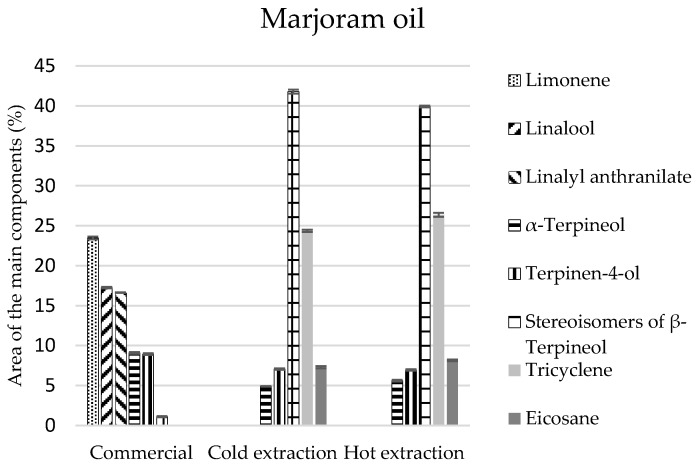
The composition of the main components in the marjoram commercial oil and extracts obtained in the process of cold and hot extraction.

As for marjoram oils, the greatest number of compounds was identified in the commercial oil (21 components), while in the extract there were 15 components for cold and 12 components for hot extraction (Table 5). The chemical analysis of these oils revealed that they are mainly composed of monoterpenes and monoterpenoids. In the commercial oil their composition was similar, and equalled 40%. In the oils obtained by extraction methods, the percentage of monoterpenes was 26–27%, and the amount of monoterpenoids was two times larger and equalled 55% (Table 5). Limonene constituted the most abundant component of the commercial oil (at least 23%) (Figure 4), and *β*-terpineol stereoisomers were present in the extracts (approximately 40% of the composition), which were present in the commercial oil in the amount of 1% (Figure 4).

**Figure 5:**
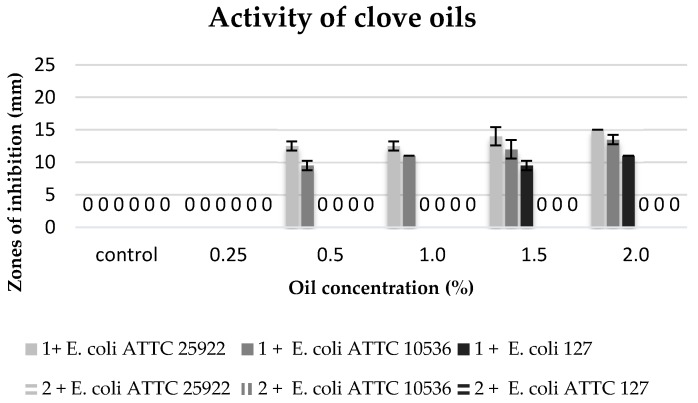
Zones of inhibition of the tested *E. coli* strains against the clove oils. 1: commercial oil; 2: cold extract.

**Figure 6:**
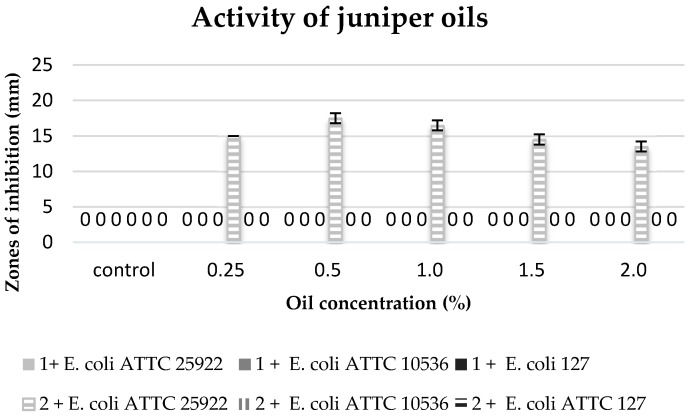
Zones of inhibition of the tested *E. coli* strains against the juniper oils. 1: commercial oil; 2: cold extract.

**Figure 7:**
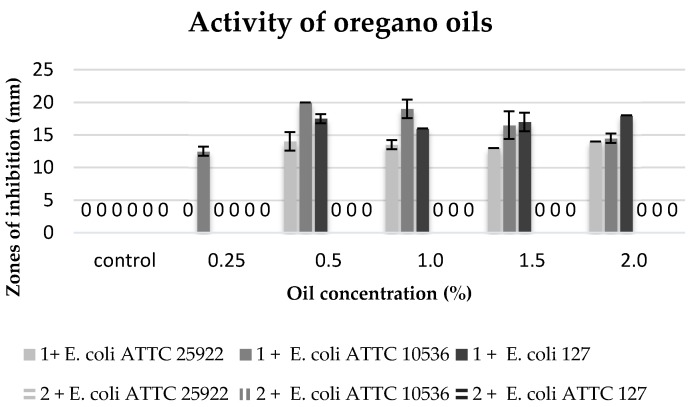
Zones of inhibition of the tested *E. coli* strains against the oregano oils. 1: commercial oil; 2: cold extract.

**Figure 8:**
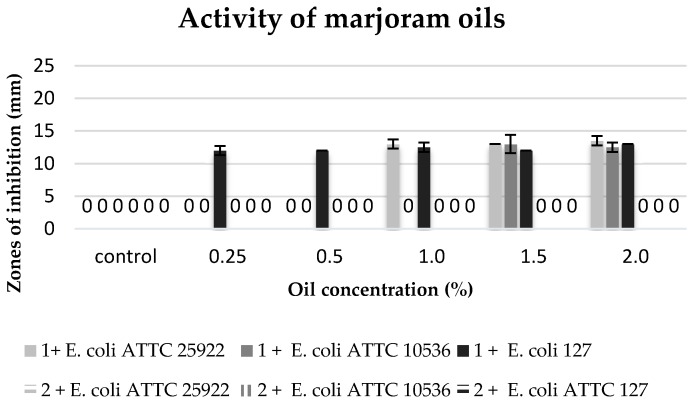
Zones of inhibition of the tested *E. coli* strains against the marjoram oils. 1: commercial oil; 2: cold extract.

The biological activity of commercial oils and extracts obtained in the process of cold and hot maceration against three strains of *E. coli* was differentiated (Table 6, Figure 5, Figure 6, Figure 7 and Figure 8).

The degree of growth inhibition of the tested bacterial strains depended both on the concentration and the chemical composition related to the methods of obtaining oils (Table 6).

The commercial clove oil with the concentration of 0.5–2% inhibited the growth of the stains in the collection (*E. coli* ATTC 25922 and *E. coli* ATTC 10536). The *E. coli* ATTC 25922 strain proved to be more sensitive, and the growth inhibition zones were 12.5 to 15 mm. On the other hand, the environmental isolate *E. coli* 127 revealed sensitivity only to higher concentrations of this oil (1.5–2%). However, no biocidal effect was observed of clove extracts against the tested bacterial strains (Figure 5).

The commercial juniper oil did not reveal any antibacterial properties against any of the tested bacterial strains, and the extract obtained in the process of maceration only inhibited the growth of the strain of *E. coli* 25922 at all used concentrations of this extract (Figure 6).

The commercial oregano oil inhibited the growth of all the tested strains of bacteria *E. coli* in the concentrations ranging from 0.5% to 2%, yet, the maximum zone of inhibition bacteria growth was present at concentrations of 0.5% and 1%. The *E. coli* ATTC 10536 strain proved to be the most sensitive to the oil activity. However, no biocidal activity was observed of clove macerate against the tested bacterial strains (Figure 7).

The commercial marjoram oil, within the range of concentration 1.5–2%, inhibited the growth of the tested *E. coli* strains. It revealed a similar inhibitory activity against the strains of *E. coli* ATTC 25922 and *E. coli* 127. *E. coli* ATTC 10536 showed a higher sensitivity to this oil (Figure 8).

## 3. Discussion

Essential oils show a differentiated chemical composition, which depends on various factors, such as the source of the raw material, climate conditions of plantations, the methods of obtaining the oils, etc. [23,24,25,26]. The tested commercial essential oils revealed considerable differences in terms of the composition and diversity of terpenes, terpenoids, and sesquiterpenes as compared with the extracts obtained from plant material. The clove, juniper, and marjoram oils contained a lower number of components as compared with their commercial counterparts, which may be caused by the loss of high-volatility compounds in the process of drying of the plant material used as the raw material for extraction [27]. On the other hand, the choice of extraction method mainly affected the quantitative composition of the oils, e.g., a higher share of sesquiterpenes was observed as compared with monoterpenes, which also may result from the nature of the raw material used for extraction purposes.

The commercial clove, oregano, and marjoram oils in the concentrations ranging from 1.5% to 2% showed antibacterial properties against all the tested strains of *E. coli*. However, these strains were not sensitive to essential oils obtained from the plant material in the process of maceration.

This suggests that the tested strains of *E. coli* show a high sensitivity mainly against monoterpenes, such as *α*-pinene, *β*-pinene, *α*,*β*,*γ*-terpinene, limonene, and some terpenoids (thymol, carvacrol). Hajlaoui et al. [28] linked the antimicrobial properties of marjoram essential oil with the high proportion of oxygenated monoterpenes, such as terpinen-4-ol, *α*-terpinol, *α*-pinene, and *p*-cymene. Additionally, the oxygenated compound, especially oxygenated monoterpenes and phenylpropanoids, might be responsible for the antimicrobial activity of marjoram oil against *Clostridium perfringens* [29].

Other authors also connect the antibacterial properties of essential oils with the presence of monoterpenes. For instance, Król et al. [30] and Dorman and Deans [31] link the activity of clove oil with the presence of eugenol, *β*-pinene, and *β*-terpinene. Additionally, de Oliveira et al. [32] showed in their study that eugenol is responsible for the phytotoxic activity of clove essential oil. On the other hand, Fun and Baerheim [33] claim that the activity of marjoram oil is induced by limonene (23.5%), *α*,*β*-pinene and *γ*-terpinene, and that a high concentration of monoterpene tricyclene leads to the reduction of the antibacterial activity of this oil. The latter statement is confirmed by the results of our own study, since the marjoram obtained in the extraction process with a high concentration of tricyclene (24%) did not inhibit the growth of any tested strain of *E. coli*.

Furthermore, the high anti-microbiological activity of the oregano oil is connected with the high concentration of terpenoids, carvacrol in particular [17,18,34]. However, the tested commercial oregano oil did not contain carvacrol, but only thymol (11%), *p*-thymol (33%), and limonene (15.5%). Most probably, it was these compounds present in high concentrations that were responsible for the inhibition of *E. coli*. The oil obtained in the process of maceration, despite the fact it contained carvacrol (1.8%) and thymol (2.5%), probably did not reveal any biological activity due to the low concentrations of these compounds.

A different reaction of bacteria *E. coli* was observed against the commercial juniper oil. These strains did not show sensitivity to the components of this oil; however, they were sensitive to the action of the obtained juniper extracts. The bactericidal activity of juniper extracts against the strains of *E. coli* is most probably connected with the presence of sesquiterpenes which were not isolated from the commercial essential oil. The commercial juniper oil contained mainly terpenes (88%) and terpenoids (8%), and the extracts (depending on the applied procedure) contained lower amounts of terpenes (42–48%) and terpenoids (approximately 1%), yet high amounts of sesquiterpenes (25–30%). The anti-microbiotic properties of the juniper oil obtained in the maceration process seem to be caused by the synergistic activity of mono- and sesquiterpenes, which was also observed in the case of the activity of coriander oil against yeasts *Candida albicans* [35]. The antioxidant activity of juniper berry oil from Bulgaria against *Saccharomyces cerevisiae* depended on monoterpenes, mostly from α-pinene as main component [36]. The biological activity of the tested essential oils not only depends on the presence of active compounds, but also on the sensitivity of the tested strains of *E. coli*. 

## 4. Materials and Methods

### 4.1. Plant Material

The scientific material consisted in the commercial oils: clove (*Eugenia caryophyllus*) leaf oil from Etja, Elbląg, Poland; juniper (*Juniperus communis*) oil from Etja, Elbląg, Poland; oregano (*Origanum vulgare*) leaf oil from Aromatika, Kiev, Ukraine; and marjoram (*Majorana hortensis*) oil from Profarm, Lębork, Poland. The dried clove flower bud *Syzygium aromaticum* from Prymat, Jastrzębie-Zdrój, Poland; juniper berries (*Juniperus communis*) from Kawon-Hurt, Gostyń, Poland; dried marjoram leaves *Majorana hortensis* from Kawon-Hurt, Gostyń, Poland; and dried oregano leaves (*Origanum vulgare*) from Dary Natury, Grodzisk, Poland were used to obtain the appropriate herbal extracts in the process of cold and hot maceration in the Soxhlet apparatus.

### 4.2. Bacterial Strains

The biological activity of all the studied essential oils was assessed against three *E. coli* strains—*E. coli* ATTC 25922, *E. coli* ATTC 10536, and *E. coli* 127 isolated from poultry waste (strains came from the collection of the Independent Department of Biotechnology and Molecular Biology, University of Opole, Opole, Poland).

### 4.3. Study Methods

#### 4.3.1. Physicochemical

(a) Maceration—Twenty grams of dry plant material was minced in a mortar and ground with 100 cm^3^ of dichloromethane. The mixture was percolated under reduced pressure. Next, anhydrous magnesium sulphate was added to the solution which was percolated again. The solution was placed in the weighed round-bottom flask and concentrated in the rotary evaporator Heidolph Laborota 4000 efficient (Heidolph Instruments GmbH & Co. KG, Schwabach, Germany). The cooled flask was weighed again, and the extraction efficiency was calculated (%).
(b) Extraction in the Soxhlet apparatus—Fifteen grams of dry plant material was minced in a mortar and moved to the thimble, which was placed in the Soxhlet apparatus. Dichloromethane (150 cm^3^) was poured into the round-bottom flask together with a boiling stone. The extraction set was mounted and the process was carried out for 4 h. After cooling, anhydrous magnesium sulphate was added to the solution, which was then percolated under reduced pressure. The solution was placed in the weighed flask and the solvent was evaporated in the rotary evaporator. The cooled flask was weighed again and the extraction efficiency was calculated (in %).

#### 4.3.2. The Gas Chromatography Mass Spectrometry Analysis

The Hewlett Packard HP 6890 series GC system chromatograph (Hewlett Packard, Waldbronn, Germany) was used for the study, which was coupled with the Hewlett Packard 5973 mass selective detector (Hewlett Packard, Waldbronn, Germany) The chromatograph was equipped with the non-polar, high-temperature ZB-5HT capillary column (length, 30 m; inner diameter, 0.32 mm; film thickness, 0.25 μm, Phenomenex Inc., Torrance, California, USA). The on-column injector was used and 1 μm of a sample was introduced. The results of carrying out the process: initial temperatures, both of the injector and the oven were 60 °C, and the temperature was increased by 10 °C per minute up to 280 °C, the auxiliary temperature was 300 °C. Helium was used as the carrier gas and its flow was 2 mL/min. The components were identified by comparison of their mass spectra with the spectrometer database of the NIST 11 Library (National Institute of Standards and Technology, Gaithersburg, MD, USA). Each chromatographic analysis was repeated three times. The average value of relative composition of essential oil percentage were calculated from the peak areas.

#### 4.3.3. Biological

The assessment of biological activity of commercial oils and macerates against the growth of three tested *E. coli* strains was carried out by the diffusion cylinder-plate method on the Nutrient Lab-Agar^TM^ medium [5]. The media were inoculated with 1 cm^3^ of standard bacterial suspension with the optical density of ζ = 2 and the wavelength of 550 nm. The results were presented as a mean value of the growth inhibition diameter (in mm). The inhibition effect was assumed to be the lack of growth around wells, growth stimulation–intensified growth around wells and the neutral effect–growth inhibition at the edges of the wells. The control was water with 0.05% Tween 80. Essential oils and extract were used in the following concentrations: 0.25%, 0.5%, 1%, 1.5% and 2% (*v*/*v*). Each experiment was repeated four times.

## 5. Conclusions

Commercial essential oils and oils obtained in the process of extraction from plant material differed in terms of the content and the quantitative ratio of terpenes, terpenoids, and sesquiterpenes, as well as their antibacterial activity.

The commercial clove, oregano, and marjoram oils in concentrations of 1.5–2% revealed the antibacterial activity against all tested *E. coli* strains.

Essential oils obtained from plant material in the process of maceration did not reveal bactericidal activity, except for the juniper oil.

The bactericidal activity of the juniper extract against *E. coli* strains is probably related to presence of sesquiterpenes which were not isolated from the commercial essential oil.

## Figures and Tables

**Table 1:** The extraction efficacy of the tested plant raw material.

Oil	Extraction Efficacy (%)	
	Cold Extraction—Maceration	Hot Extraction—Soxhlet Apparatus
Clove	20.3	20.6
Juniper	13.1	12.4
Oregano	4.0	3.3
Marjoram	12.2	12.6

**Table 2:** Comparison of the composition of particular compounds in clove oils.

Compound	Area (%) ± Standard Deviation (SD)		
	Commercial	Maceration	Soxhlet Apparatus
**Monocyclic monoterpenes**			
*β*-Phellandrene	0.07 ± 0.01	-	-
*α*-Phellandrene	0.08 ± 0.00	-	-
*α*-Terpinene	0.02 ± 0.01	-	-
*o*-Cymene	0.66 ± 0.02	-	-
*γ*-Terpinene	0.36 ± 0.04	-	-
Terpinolene	0.03 ± 0.01	-	-
**Bi and tricyclic monoterpenes**			
*α*-Pinene	0.27 ± 0.01	-	-
Camphene	0.05 ± 0.01	-	-
*β*-Pinene	0.05 ± 0.01	-	-
Sum	1.56	-	-
**Monocyclic monoterpenoids**			
Menthol	0.02 ± 0.01	-	-
Eugenol	61.94 ± 0.23	54.16 ± 0.61	54.69 ± 0.21
Eugenyl acetate	4.13 ± 0.13	23.49 ± 0.61	27.65 ± 0.12
**Bi and tricyclic monoterpenoids**			
1,8-cineole	12.07 ± 0.46	-	0.05 ± 0.007
Bornyl acetate	0.08 ± 0.02	-	-
Sum	78.24	77.65	82.39
**Aliphatic sesquiterpenes**			
*α* -Farnesene	-	0.16 ± 0.01	-
**Monocyclic sesquiterpenes**			
*α*-Humulene	3.03 ± 0.03	1.85 ± 0.04	1.41 ± 0.02
*α*-Elemene	-	0.23 ± 0.02	0.14 ± 0.01
**Bi and tricyclic sesquiterpenes**			
*α*-Cubebene	0.10 ± 0.01	0.71 ± 0.04	0.56 ± 0.03
*β*-Cubebene	-	0.34 ± 0.02	0.24 ± 0.02
*β*-Caryophyllene	15.86 ± 0.19	15.76 ± 0.38	13.11 ± 0.33
*α*-Copaene	-	2.00 ± 0.31	1.24 ± 0.22
*β*-Copaene	-		-
*γ*-Muurolene	-	0.08 ± 0.01	-
Sum	18.99	21.13	16.70
**Bi and tricyclic sesquiterpenoids**			
Cubebol	-	0.13 ± 0.02	-
Caryophyllene oxide	0.87 ± 0.02	0.40 ± 0.03	0.45 ± 0.04
Sum	0.87	0.53	0.45
**Esters**			
Methyl salicylate	0.14 ± 0.03	-	0.03 ± 0.01
**Ketones**			
2,3,4-Trimethoxyacetophenone	-	0.49 ± 0.15	0.18 ± 0.01
**Phenols**			
Chavikol	0.17 ± 0.01	0.18 ± 0.04	0.21 ± 0.03
Sum	0.31	0.67	0.42

**Table 3:** Comparison of the composition of particular compounds in juniper oils.

Compound	Area (%) ± SD		
	Commercial	Maceration	Soxhlet Apparatus
**Monocyclic monoterpenes**			
*β*-Phellandrene	12.71 ± 0.23	-	-
*α*-Phellandrene	0.48 ± 0.06	-	-
*o*-Cymene	2.46 ± 0.18	-	-
*γ*-Terpinene	3.15 ± 0.25	-	-
Terpinolene	15.39 ± 0.12	-	-
*p*-Cymene	-	0.18 ± 0.01	-
Limonene	-	3.98 ± 0.12	2.75 ± 0.01
**Bi and tricyclic monoterpenes**			
*α*-Pinene	21.79 ± 0.49	27.18 ± 0.82	26.51 ± 0.09
Camphene	4.49 ± 0.18	-	-
*β*-Pinene	4.35 ± 0.19	2.49 ± 0.10	2.32 ± 0.12
*α*-Thujene	1.42 ± 0.09		
*β*-Thujene	2.00 ± 0.14	11.01 ± 0.30	7.37 ± 0.05
4-Carene	0.28 ± 0.02	-	-
3-Carene	14.23 ± 0.19	-	-
2-Carene	2.41 ± 0.18	-	-
Sabinene	2.78 ± 0.19	3.59 ± 0.13	2.61 ± 0.07
Sum	87.94	48.43	41.56
**Monocyclic monoterpenoids**			
Terpinen-4-ol	2.34 ± 0.07	0.49 ± 0.01	0.54 ± 0.01
*α*-Terpineol	0.45 ± 0.03	-	-
Thymol methyl ether	0.28 ± 0.08	-	-
*α*-Terpineol acetate	0.52 ± 0.02	-	-
**Bi and tricyclic monoterpenoids**			
Bornyl acetete	4.04 ± 0.04	0.33 ± 0.02	0.38 ± 0.01
Borneol	0.28 ± 0.03	-	-
Thujone	0.11 ± 0.02	-	-
Fenchyl acetate	0.13 ± 0.02	-	-
Sum	8.15	0.82	0.92
**Monocyclic sesquiterpenes**			
*α*-Humulene	0.63 ± 0.03	4.13 ± 0.14	4.76 ± 0.05
*α*-Elemene	0.08 ± 0.01	-	-
*β*-Elemene	-	0.65 ± 0.03	0.80 ± 0.06
Elixene	0.06 ± 0.02	-	-
Germacrene D	0.09 ± 0.01	3.57 ± 0.54	3.85 ± 0.13
**Bi and tricyclic sesquiterpenes**			
*α*-Cubebene	-	1.43 ± 0.04	1.54 ± 0.05
*β*-Caryophyllene	1.06 ± 0.01	5.06 ± 0.17	5.24 ± 0.02
*α*-Copaene	0.05 ± 0.01	1.00 ± 0.05	1.23 ± 0.01
*α*-Muurolene	0.10 ± 0.01	0.61 ± 0.06	0.59 ± 0.01
*γ*-Muurolene	0.09 ± 0.01	0.45 ± 0.05	0.49 ± 0.01
Alloaromadendrene	-	0.46 ± 0.02	0.44 ± 0.01
*α*-Cadinene	0.16 ± 0.01	-	-
*δ*-Cadinene	0.57 ± 0.01	1.38 ± 0.03	1.80 ± 0.03
*α*-Cedrene	0.08 ± 0.01	-	-
Epizonarene	-	0.61 ± 0.01	0.96 ± 0.05
*β*-Maaliene	-	0.64 ± 0.04	-
*α*-Selinene	-	-	0.73 ± 0.03
Longifolene	0.17 ± 0.01	-	-
Sum	3.14	19.99	22.43
**Bi and tricyclic sesquiterpenoids**			
Cubebol	-	1.83 ± 0.16	1.83 ± 0.05
Caryophyllene oxide	0.19 ± 0.02	3.82 ± 0.87	4.57 ± 0.08
Spathulenol	-	-	1.40 ± 0.10
*τ*-Cadinol	0.06 ± 0.01	-	-
Sum	0.25	5.65	7.80
**Ethers**			
Estragole	0.16 ± 0.02	-	-
**Hydrocarbon derivatives**			
Cuparene	0.36 ± 0.01	-	-
Unseparated	-	25.11 ± 0.28	27.29 ± 0.21
Sum	0.52	25.11	27.29

**Table 4:** Comparison of the composition of particular compounds in oregano oils.

Compound	Area (%) ± SD		
	Commercial	Maceration	Soxhlet Apparatus
**Aliphatic monoterpenes**			
*β*-Ocimene	-	4.01 ± 0.25	4.03 ± 0.08
*α*-Ocimene	-	0.94 ± 0.11	1.14 ± 0.07
**Monocyclic monoterpenes**			
*β*-Phellandrene	-	13.69 ± 0.52	13.08 ± 0.39
*α*-Phellandrene	0.13 ± 0.01	-	-
*o*-Cymene	23.74 ± 0.30	5.98 ± 0.28	5.45 ± 0.11
*γ*-Terpinene	0.67 ± 0.02	2.04 ± 0.22	2.49 ± 0.12
Terpinolene	1.62 ± 0.05	-	-
Limonene	15.47 ± 0.62	-	-
**Bi and tricyclic monoterpenes**			
*α*-Pinene	5.90 ± 0.04	-	-
Camphene	0.80 ± 0.04	-	-
*β*-Pinene	0.09 ± 0.01	-	-
2-Carene	1.70 ± 0.08	-	-
Bornylene	1.05 ± 0.04	-	-
Sum	51.17	26.66	26.19
**Aliphatic monoterpenoids**			
Linalool	1.57 ± 0.01	-	-
**Monocyclic monoterpenoids**			
Terpinen-4-ol	0.09 ± 0.01	-	-
*α*-Terpineol	2.28 ± 0.07	-	-
*β*-Terpineol	0.36 ± 0.02	1.34 ± 0.06	1.49 ± 0.08
Thymol	11.17 ± 0.54	2.53 ± 0.15	2.13 ± 0.09
*p*-Thymol	32.69 ± 0.43	-	1.52 ± 0.04
Thymol methyl ether	-	0.63 ± 0.05	0.52 ± 0.02
Carvacrol	-	1.80 ± 0.42	-
Isothymol methyl ether	-	-	0.69 ± 0.01
**Bi and tricyclic monoterpenoids**			
1,8-cineole	-	-	1.41 ± 0.08
*endo*-Borneol	0.51 ± 0.02	-	-
*cis*-Sabinene hydrate	-	3.07 ± 0.17	2.74 ± 0.08
Sum	48.67	9.37	10.50
**Monocyclic sesquiterpenes**			
*α*-Humulene	-	1.23 ± 0.07	1.05 ± 0.05
*α*-Bisabolene	-	1.69 ± 0.06	-
**Bi and tricyclic sesquiterpenes**			
*β*-Cubebene	-	8.81 ± 0.24	7.55 ± 0.01
*β*-Caryophyllene	0.16 ± 0.01	10.51 ± 0.16	8.49 ± 0.21
*β*-Copaene	-	0.85 ± 0.03	-
*γ*-Muurolene	-	1.36 ± 0.07	1.20 ± 0.06
*β*-Burbonene	-	3.36 ± 0.10	3.14 ± 0.12
Alloaromadendrene	-	1.66 ± 0.06	1.45 ± 0.10
*δ*-Cadinene	-	1.09 ± 0.07	0.97 ± 0.03
*β*-Gurjunene	-	-	0.81 ± 0.09
Sum	0.16	30.56	24.66
**Bi and tricyclic sesquiterpenoids**			
Caryophyllene oxide	-	19.41 ± 0.15	16.15 ± 0.06
Sum	-	19.41	16.15
**Esters**			
Triacontyl acetate	-	14.51 ± 0.52	16.61 ± 0.25
**Acids**			
Linolenic acid	-	-	5.89 ± 0.25
Sum	-	14.51	22.50

**Table 5:** Comparison of the composition of particular compounds in marjoram oils.

Compound	Area (%) ± SD		
	Commercial	Maceration	Soxhlet Apparatus
**Monocyclic monoterpenes**			
*β*-Phellandrene	-	1.26 ± 0.11	-
*α*-Phellandrene	0.23 ± 0.01	-	-
*γ*-Terpinene	4.49 ± 0.09	1.16 ± 0.20	-
Terpinolene	1.10 ± 0.01	-	-
*p*-Cymene	2.40 ± 0.09	0.42 ± 0.04	-
Limonene	23.47 ± 0.18	-	-
**Bi and tricyclic monoterpenes**			
*α*-Pinene	0.71 ± 0.01	-	-
*β*-Pinene	0.66 ± 0.01	-	-
*β*-Thujene	2.07 ± 0.08	-	-
2-Carene	2.67 ± 0.05	-	-
Tricyclene	-	24.36 ± 0.15	26.37 ± 0.23
Sum	37.80	27.20	26.37
**Aliphatic monoterpenoids**			
Linalool	17.25 ± 0.08	-	-
**Monocyclic monoterpenoids**			
Eugenol	1.55 ± 0.03	-	-
*p*-Menth-2-en-1-ol	0.51 ± 0.03	1.06 ± 0.04	0.69 ± 0.01
Terpinen-4-ol	8.93 ± 0.11	7.06 ± 0.09	6.94 ± 0.09
*α*-Terpineol	9.09 ± 0.11	4.89 ± 0.04	5.63 ± 0.11
Stereoisomers of *β*-Terpineol	-	6.08 ± 0.09	4.91 ± 0.04
	1.11 ± 0.07	35.70 ± 0.17	35.01 ± 0.06
*trans*-Piperitol	-	0.56 ± 0.01	0.44 ± 0.01
**Bi and tricyclic monoterpenoids**			
Camphor	0.33 ± 0.01	-	-
Bornyl acetate	0.20 ± 0.01	-	-
Sum	38.97	55.35	53.62
**Monocyclic sesquiterpenes**			
Elixene	0.16 ± 0.01	-	-
**Bi and tricyclic sesquiterpenes**			
*β*-Caryophyllene	0.64 ± 0.02	3.89 ± 0.11	3.99 ± 0.05
Bicyclogermacrene	-	0.89 ± 0.03	0.63 ± 0.02
Sum	0.80	4.78	4.62
**Bi and tricyclic sesquiterpenoids**			
Spathulenol	-	2.19 ± 0.08	2.64 ± 0.08
Sum	-	2.19	2.64
**Diterpenes**			
*4-epi*-Dehydroabietol	-	3.31 ± 0.12	4.60 ± 0.08
Sum	-	3.31	4.60
**Esters**			
Linalyl anthranilate	16.63 ± 0.05	-	-
**Ethers**			
Estragole	5.77 ± 0.04	-	-
**Hydrocarbons**			
Eicosane	-	7.29 ± 0.15	8.15 ± 0.10
Sum	22.40	7.29	8.15

**Table 6:** The zones of inhibition of *E. coli* strains in the presence of essential oils.

Oil Concentration (%)	Zones of Inhibition (mm) ± SD					
	*E. coli* ATTC 25922		*E. coli* ATTC 10536		*E. coli* 127	
	Commercial	Maceration	Commercial	Maceration	Commercial	Maceration
**Clove oil**						
0.25	0	0	0	0	0	0
0.5	12.5 ± 0.7	0	9.5 ± 0.7	0	0	0
1.0	12.5 ± 0.7	0	11.0 ± 0.0	0	0	0
1.5	14.0 ± 1.4	0	12.0 ± 1.4	0	9.5 ± 0.7	0
2.0	15.0 ± 0.0	0	13.5 ± 0.7	0	11.0 ± 0.0	0
**Juniper oil**						
0.25	0	15.0 ± 0.0	0	0	0	0
0.5	0	17.5 ± 0.7	0	0	0	0
1.0	0	16.5 ± 0.7	0	0	0	0
1.5	0	14.5 ± 0.7	0	0	0	0
2.0	0	13.5 ± 0.7	0	0	0	0
**Oregano oil**						
0.25	0	0	12.5 ± 0.7	0	0	0
0.5	14.0 ± 1.4	0	20.0 ± 0.0	0	17.5 ± 0.7	0
1.0	13.5 ± 0.7	0	19.0 ± 1.4	0	16.0 ± 0.0	0
1.5	13.0 ± 0.0	0	16.5 ± 2.1	0	17.0 ± 1.4	0
2.0	14.0 ± 0.0	0	14.5 ± 0.7	0	18.0 ± 0.0	0
**Marjoram oil**						
0.25	0	0	0	0	7.5 ± 10.6	0
0.5	0	0	0	0	12.0 ± 0.0	0
1.0	6.5 ± 9.2	0	0	0	12.5 ± 0.7	0
1.5	13.0 ± 0.0	0	13.0 ± 1.4	0	12.0 ± 0.0	0
2.0	13.5 ± 0.7	0	12.5 ± 0.7	0	13.0 ± 0.0	0
